# Risk factors for high-grade pivot shift in patients with anterior cruciate ligament tears combined with lateral meniscus posterior root tears: a systematic review and meta-analysis

**DOI:** 10.3389/fmed.2026.1813111

**Published:** 2026-05-07

**Authors:** Peng Zhang, Xiao Wang, Xiaotao Shi, Yanhao Yuan, Dongdong Xie, Feilong Du, Yisan Wang, Mingyang Zhang, Honglüe Tan, Guorui Cao

**Affiliations:** 1 Hunan University of Chinese Medicine, Changsha, China; 2 Department of Knee Surgery, Luoyang Orthopedic Hospital of Henan Province, Orthopedic Hospital of Henan Province, Luoyang, Henan, China; 3 Fujian University of Traditional Chinese Medicine, Fuzhou, China; 4 School of Orthopedics and Traumatology, Henan University of Chinese Medicine, Zhengzhou, China

**Keywords:** ACL injury, lateral meniscus posterior root tear, pivot shift, rotational instability, the incidence

## Abstract

**Objective:**

High-grade pivot shift is the most common complication in patients with anterior cruciate ligament (ACL) tears combined with lateral meniscus posterior root tears (LMPRT), and it significantly impairs quality of life. Risk factors for high-grade pivot shift in this patient population have not been fully investigated. This meta-analysis aims to evaluate the incidence and risk factors of high-grade pivot shift in these patients to provide evidence-based guidance for clinical treatment.

**Methods:**

We searched for observational studies investigating high-grade pivot shift in patients with ACL tears combined with LMPRT in PubMed, Embase, Cochrane Library, Web of Science, CNKI, Wanfang, VIP, and SinoMed from the inception of each database through November 2025, using a combination of MeSH terms and free-text keywords. Basic study characteristics, incidence of high-grade pivot shift, and relevant risk factors were extracted from included studies. Methodological quality and risk of bias were assessed, and meta-analysis of incidence rates and risk factors was performed using Stata 18.0 software.

**Results:**

A total of twenty studies involving 8,102 patients were included. High-grade pivot shift was identified in 46% of patients with isolated ACL tears (95% *CI*: 0.26, 0.65) and in 72% of those with ACL tears combined with LMPRT (95% *CI*: 0.54, 0.89). Nineteen of the included studies were rated as high quality. Younger age (*MD* = −0.13, 95% *CI*: −0.19 to −0.06), elevated KT-SSD (*MD* = 0.77, 95%*CI*: 0.28–1.26; *OR* = 5.13, 95%*CI*: 3.01–8.74), increased anterior tibial translation (*MD* = 0.90, 95%*CI*: 0.23 to 1.58), joint laxity (*OR* = 1.78, 95%*CI*: 1.03–3.08), severe meniscal extrusion (*MD* = 1.51, 95%*CI*: 0.52–2.49), increased lateral tibial slope (*MD* = 0.15, 95%*CI*: 0.03–0.26), and complete meniscal tear (*OR* = 5.01, 95%*CI*: 2.89–8.66) were independent risk factors for high-grade pivot shift in patients with ACL tear combined with LMPRT.

**Conclusion:**

Younger age, elevated KT-SSD values, increased anterior tibial translation, joint laxity, severe meniscal extrusion, increased lateral tibial slope, and complete meniscal tear are independent risk factors for high-grade pivot shift in patients with ACL tear combined with LMPRT. Patients with these high-risk factors require enhanced follow-up and timely intervention to reduce the incidence of high-grade pivot shift and improve patients’ quality of life.

**Systematic review registration:**

https://www.crd.york.ac.uk/PROSPERO/home, identifier CRD420251269936.

## Introduction

Anterior cruciate ligament (ACL) tears are a common sports-related knee joint injury. They remain prevalent among young, physically active individuals and often lead to damage of adjacent structures such as the meniscus and collateral ligaments, which significantly compromises knee joint stability and functional recovery.(1) Lateral meniscus posterior root tears (LMPRT) occur in approximately 10–15% of patients with ACL tears and represent tears at the attachment site of the meniscal posterior root.(2) This injury disrupts the circumferential stress mechanism of the meniscus, rendering it ineffective in restricting anterior tibial translation and rotation. Consequently, it exacerbates anterolateral rotational instability of the knee joint, resulting in a dual-instability pathological state when combined with an ACL tear.(3)

The pivot-shift test is a key clinical sign for assessing rotational instability of the knee joint. In addition to significantly impairing patients’ preoperative functional mobility and quality of life, high-grade pivot shift increases the risk of graft failure, secondary injury, and knee osteoarthritis following ACL reconstruction.(4) Previous studies have shown that younger age, male sex, and meniscal extrusion are risk factors strongly associated with an increased incidence of high-grade pivot shift in patients with ACL tears combined with LMPRT. However, several research findings remain controversial, and there is a paucity of robust, well-designed studies.(5)

Currently, there is insufficient clinical evidence regarding the risk factors for high-grade pivot shift in patients with ACL tears combined with LMPRT, and no study has comprehensively investigated this topic. Therefore, this study employed a systematic review and meta-analysis to explore potential risk factors for high-grade pivot shift in these patients. The aim is to provide more reliable and precise results, as well as evidence-based support for the surgical management of this patient population.

## Materials and methods

This systematic review and meta-analysis was reported in accordance with the Preferred Reporting Items for Systematic Reviews and Meta-Analyses (PRISMA) guidelines.(6) This study was registered in PROSPERO (registration number: CRD420251269936).

### Search strategy

Electronic searches were performed across PubMed, Embase, Cochrane Library, Web of Science, CNKI, Wanfang, VIP, and SinoMed from database inception through November 2025. The search strategy employed the following key terms: (((anterior cruciate ligament) AND (lateral meniscus OR lateral meniscus posterior root OR posterior root injury of the lateral meniscus)) AND (pivot-shift test OR axial displacement test OR tibial axial displacement OR pivot shift OR high-grade pivot shift)) AND (risk factors OR cohort studies). Both free-text words and controlled vocabulary (MeSH terms) were used. Additional searches were manually conducted by screening the reference lists of included studies and relevant reviews.

### Study selection

Studies were eligible for inclusion if they met the following criteria: (1) reported quantitative data on risk factors for high-grade pivot shift in patients with ACL tears combined with LMPRT; (2) investigated at least one risk factor associated with ACL tears combined with LMPRT; (3) used an observational study design, including cross-sectional, case-control, prospective cohort, or retrospective cohort studies; (4) enrolled only patients who underwent ACL reconstruction; (5) were published as full-text articles; (6) used the International Knee Documentation Committee (IKDC) grading system to define high-grade pivot shift (either the classic 4-grade or modified 6-grade scale); (7) confirmed LMPRT diagnosis via magnetic resonance imaging (MRI) or arthroscopic inspection.

Exclusion criteria included animal or cadaveric studies, case series, narrative reviews, systematic reviews, editorials, commentaries, and opinion articles. Discrepancies between the two independent reviewers regarding study eligibility were resolved by a third reviewer.

### Data extraction

Two reviewers independently extracted the following data using a standardized electronic form: first author, year of publication, study design, study population, country, participant demographics (age, sex), prevalence of ACL tears combined with LMPRT, diagnostic methods, and relevant risk factors. Any disagreements were resolved through discussion with the third reviewer.

### Quality assessment

Two independent reviewers assessed the quality of the included studies using the Newcastle–Ottawa Scale (NOS).(7) The NOS comprises eight items across three domains: selection (four items, 1 point each), comparability (1 domain, up to 2 points), and outcome (three items, 1 point each). Studies scoring 7 or more points were classified as high-quality. Any discrepancies between the two independent reviewers were resolved via discussion with a third reviewer.

### Statistical analysis

Statistical analyses were performed using Stata 18.0 software. Pooled odds ratios (ORs) with corresponding 95% confidence intervals (CIs) were calculated for each risk factor. Between-study heterogeneity was evaluated using the Cochran Q test and *I*^2^ statistic. A *P*-value < 0.10 indicated significant heterogeneity. Heterogeneity was graded as follows: *I*^2^ < 25% for low heterogeneity, 25% ≤ *I*^2^ < 50% for moderate heterogeneity, and *I*^2^ ≥ 50% for high heterogeneity. A fixed-effect model was applied when *P* > 0.10 and *I*^2^ < 50%, indicating low heterogeneity. A random-effects model was used when *P* < 0.10 or *I*^2^ ≥ 50%, indicating substantial heterogeneity. If significant heterogeneity was detected, sensitivity and subgroup analyses were performed to identify potential sources. A *P*-value < 0.05 was considered statistically significant. Publication bias was assessed using funnel plots and Egger’s test; a *P*-value > 0.05 from Egger’s test indicated no significant publication bias.

## Results

### Search results

**FIGURE 1:**
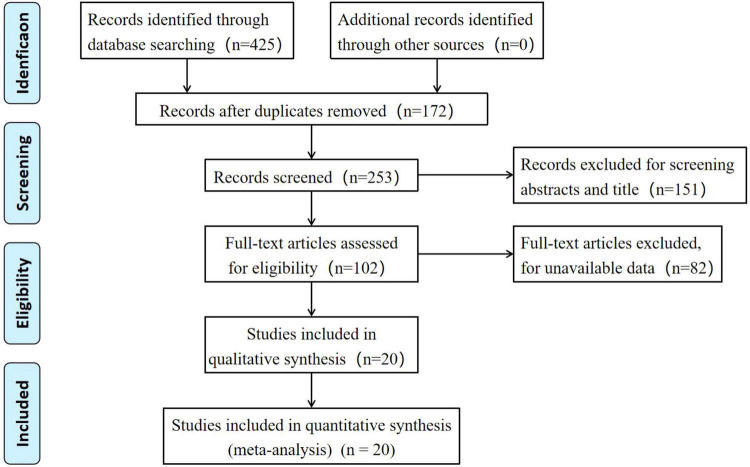
Flowchart of retrieval process according to PRISMA statement.

The initial database search identified 425 potential records. After removal of 172 duplicates, 253 articles were screened by title and abstract, of which 151 irrelevant studies were excluded. Full texts were obtained for the remaining 102 articles, and a further 82 articles were excluded based on the predefined inclusion and exclusion criteria. Finally, twenty studies (1, 2, 5, 8–23) were included in this meta-analysis. The study selection process is illustrated in Figure 1.

### Characteristics of included studies

Table 1 summarizes the baseline characteristics of the included studies. A total of 8,102 patients were enrolled across the twenty studies, and the mean age of patients with ACL tears combined with LMPRT was 27.07 years. Of the twenty included studies, eleven reported the incidence of high-grade pivot shift. Publication years ranged from 2017 to 2025, with only four studies (2, 8, 9, 20) published before 2020. Seventeen studies were conducted in Asia (three in Japan, twelve in China, and two in South Korea), two in Europe (one in France and one in Sweden), and one in North America (the United States).

**TABLE 1:** Characteristics of included studies.

No.	First author (year)	Country	Study design	No. of patients		Age (years)		Sex (male/ female, %)	High grade of pivot shift		NOS score	References
				LMPR+ (n)	LMPR- (n)	LMPR+ (n)	LMPR- (n)		LMPR+ (n/%)	LMPR- (n/%)		
1	Tong 2019(a)	China	Case-Control	45	51	30.0 ± 9.2	30.2 ± 8.7	77.08/22.92	31/68.89	9/17.65	7	(9)
2	Tong 2019(b)	China	Case-Control	60	26	27.2 ± 7.9	29.3 ± 9.2	76.74/23.26	NR	NR	8	(8)
3	Zan 2025	China	Cohort	52	200	24.10 ± 8.10	29.60 ± 8.80	66.27/33.73	NR	NR	8	(10)
4	Jiachun 2022	China	Case-Control	48	22	27.32 ± 7.65	29.61 ± 7.92	75.71/24.29	NR	NR	7	(11)
5	Zhihang 2020	China	Case-Control	84	89	26.7 ± 7.29	31.5 ± 9.49	79.77/20.23	NR	NR	8	(12)
6	Guanyang 2025	China	Cohort	41	129	29.8 ± 9.6	32.3 ± 11.4	55.88/44.12	38/92.68	64/49.61	8	(23)
7	Bo 2024(a)	China	Cohort	10	18	32.00 ± 10.34	36.56 ± 11.45	60.71/39.29	NR	NR	7	(13)
8	Bo 2024(b)	China	Cohort	17	18	32.41 ± 10.97	36.56 ± 11.45	40.00/60.00	NR	NR	7	(13)
9	Qiankun 2022	China	Case-Control	35	36	27.0 ± 9.2	29.6 ± 8.3	77.46/22.54	25/71.43	13/36.11	9	(14)
10	Seong Hwan 2020	Korea	Cohort	31	89	29.3 ± 8.3	31.1 ± 7.9	84.17/15.83	21/67.74	36/40.45	7	(15)
11	Zipeng 2023	China	Case-Control	113	113	29.1 ± 7.8	29.3 ± 7.9	70.80/29.2	104/92.04	56/49.56	9	(16)
12	Aritoshi 2023	Japan, Luxembourg	Case-Control	126	126	26.0 ± 10.37	26.0 ± 9.63	58.73/41.27	102/80.95	86/68.25	8	(17)
13	Seong Hwan 2022	Korea	Cohort	22	204	28.0 ± 11.3	30.5 ± 11.3	84.96/15.04	18/81.82	82/40.20	7	(18)
14	Takao 2018	Japan	Case-Control	39	117	24.0 ± 12.5	25.0 ± 10.0	50.64/49.36	27/69.23	77/65.81	7	(2)
15	Weiding 2021	Japan	Case-Control	265	101	25.0 ± 10.0	27.4 ± 10.9	51.37/48.63	55/20.75	7/6.93	7	(19)
16	Gian Andrea 2024	USA	Cohort	22	77	19.0 ± 11.85	21.0 ± 10.37	54.55/45.45	14/63.64	37/48.05	7	(22)
17	Chengpang 2024	China	Case-Control	33	19	24.4 ± 7.9	25.9 ± 10.7	51.92/48.08	NR	NR	6	(21)
18	Pierre-Jean 2024	France	Case-Control	375	4984	28.7 ± 10.5	29.4 ± 10.3	62.74/37.26	289/77.07	3915/78.55	8	(5)
19	Guanyang 2017	China	Case-Control	51	23	27.4 ± 8.4	29.2 ± 8.1	75.68/24.32	NR	NR	7	(20)
20	Riccardo 2024	Sweden	Cohort	149	42	26.4 ± 7.4	25.1 ± 7.3	47.12/52.88	NR	NR	8	(1)

### Quality assessment

Methodological quality scores for the included studies are presented in Table 1. Of the twenty studies, nineteen were rated as high quality, and only one study (21) was of moderate quality.

### Incidence of high-grade pivot-shift

**FIGURE 2:**
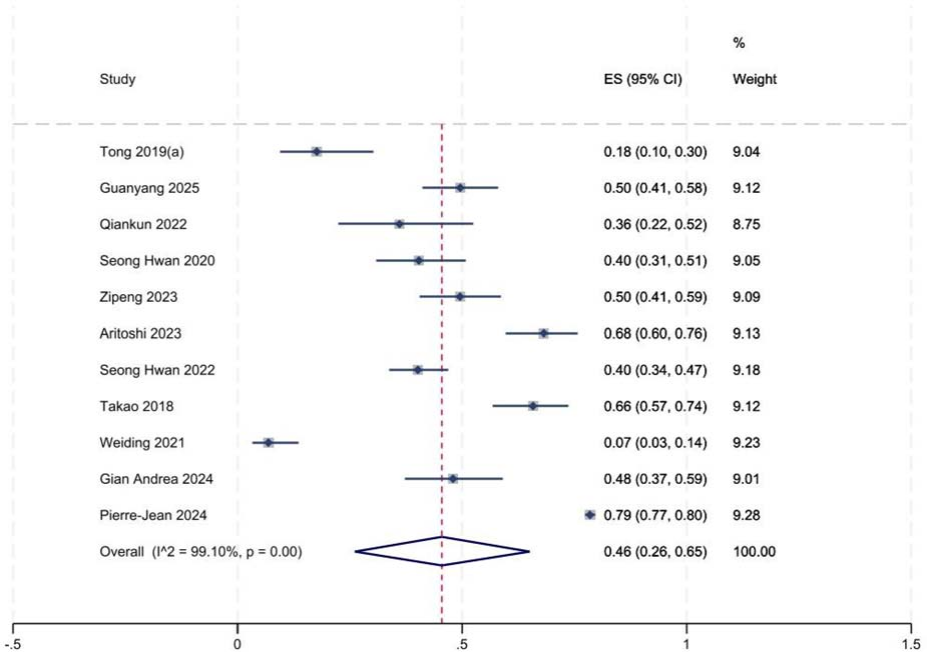
Forest plot of high-grade pivot shift incidence (LMPR−).

**FIGURE 3:**
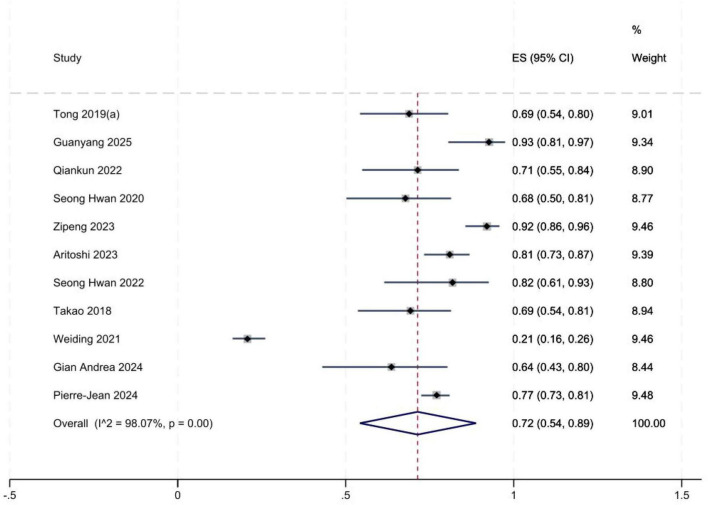
Forest plot of high-grade pivot shift incidence (LMPR+).

**FIGURE 4:**
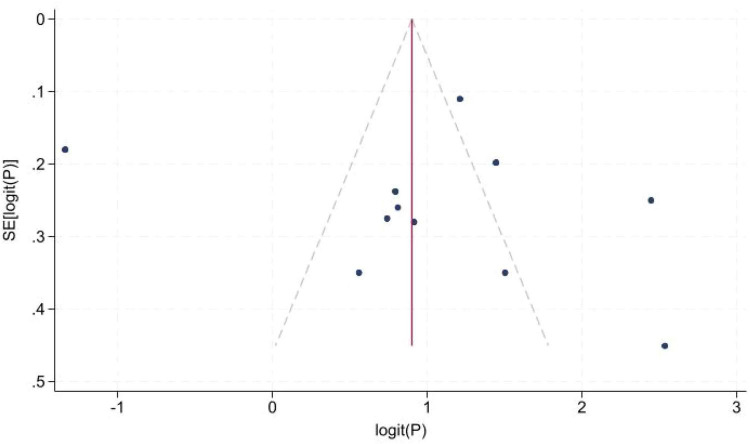
Publication bias of the incidence of high-grade pivot shift.

A total of 11 studies (2, 5, 8, 14–19, 22, 23) reported the incidence of high-grade pivot shift. Significant between-study heterogeneity was observed. Sensitivity analyses were performed to explore potential sources of heterogeneity; no substantial changes in pooled estimates were identified after omitting any single study. Meta-analysis showed that the incidence of high-grade pivot shift was 46% (95% *CI*: 0.26, 0.65) in patients with isolated ACL tears (Figure 2) and 72% (95% *CI*: 0.54, 0.89) in those with ACL tears combined with LMPRT (Figure 3). Publication bias was evaluated using funnel plots and Egger’s test (Figure 4). Egger’s test yielded a *P*-value of 0.723 ( > 0.05), indicating no significant publication bias across the included studies.

### Risk factors

#### Demographic risk factors

##### Age

**FIGURE 5:**
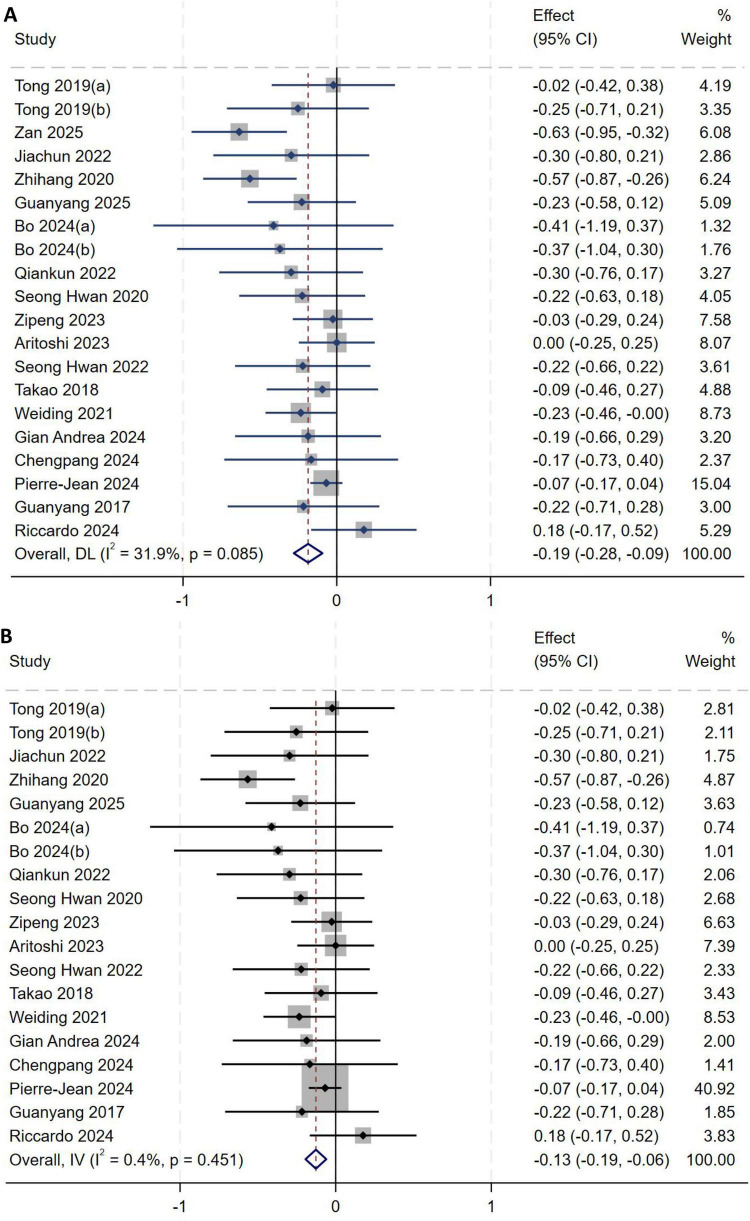
**(A)** Initial analysis with moderate heterogeneity. **(B)** Sensitivity analysis after excluding the study by Zan et al., showing the pooled effect size.

A total of twenty studies (1, 2, 5, 8–23) included age in their analysis. Heterogeneity testing revealed an *I*^2^ of 31.9% (*P* = 0.085), indicating moderate between-study heterogeneity, as shown in Figure 5A. A sensitivity analysis with stepwise omission was performed to identify the source of heterogeneity. Exclusion of the study by Zan et al. (10) markedly reduced heterogeneity (*I*^2^ = 0.4%, *P* = 0.451). A fixed-effect model was therefore used for the meta-analysis. The pooled results demonstrated that younger age was associated with an increased risk of high-grade pivot shift (*MD* = −0.13, 95% *CI*: −0.19 to −0.06, *P* < 0.01), as shown in Figure 5B.

##### Sex

**FIGURE 6:**
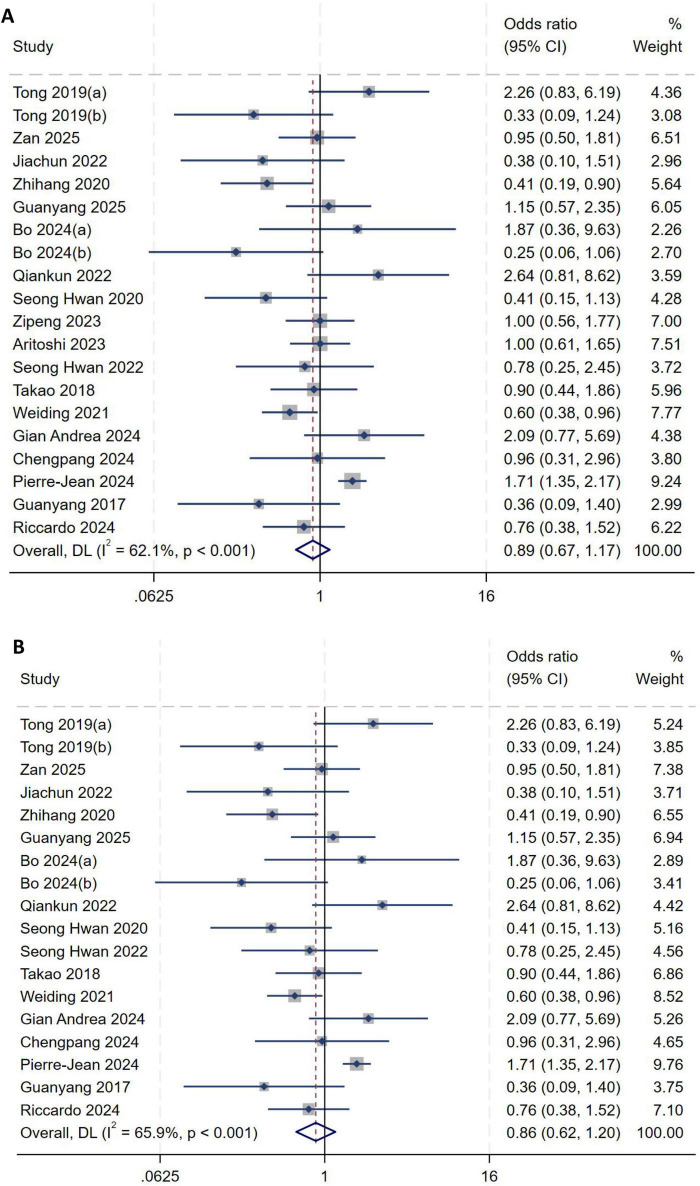
**(A)** Initial analysis with substantial heterogeneity. **(B)** Subgroup analysis after excluding the matched subgroup, showing the pooled effect size.

A total of twenty studies (1, 2, 5, 8–23) included sex in their analysis. Heterogeneity testing showed an *I*^2^ of 62.1% (*P* < 0.001), indicating substantial between-study heterogeneity, as shown in Figure 6A. Sensitivity analysis did not significantly alter the overall results. A subgroup analysis was performed based on whether studies used a 1:1 matching ratio to further explore sources of heterogeneity. However, heterogeneity remained high because only two studies employed this 1:1 ratio. In addition, results in the LMPRT-negative group may not have accurately reflected the true incidence, as this group was strictly matched to the LMPRT-positive group. Consequently, this subgroup was excluded from the final analysis, and a random-effects model was used to pool effect sizes. The pooled results demonstrated no significant association between sex and the incidence of LMPRT after ACL reconstruction (*OR* = 0.86, 95%*CI*: 0.62–1.20, *P* = 0.50), as shown in Figure 6B.

##### Body mass index (BMI)

**FIGURE 7:**
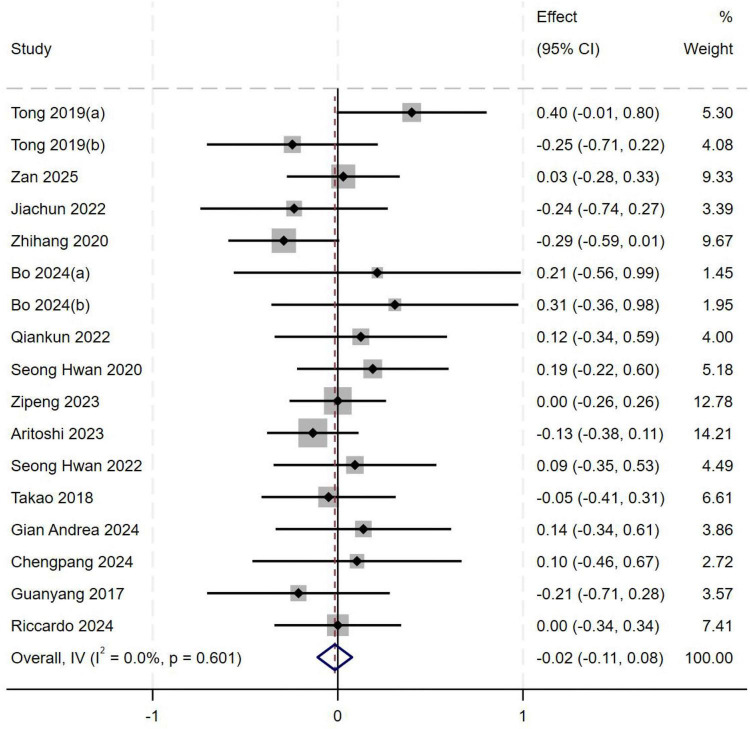
Forest plot of BMI in LMPR+ group versus LMPR− group.

A total of seventeen studies (1, 2, 8–18, 20–22) were included in the analysis of BMI. Heterogeneity testing showed an *I*^2^ of 0% (*P* = 0.601), indicating no significant between-study heterogeneity. A fixed-effect model was therefore used to pool effect sizes. Meta-analysis demonstrated no statistically significant association between BMI and the occurrence of high-grade pivot shift (*MD* = −0.02, 95%*CI*: −0.11 to 0.08, *P* = 0.734), as shown in Figure 7.

#### Preoperative joint function and stability risk factors

##### Lachman test (pivot height)

**FIGURE 8:**
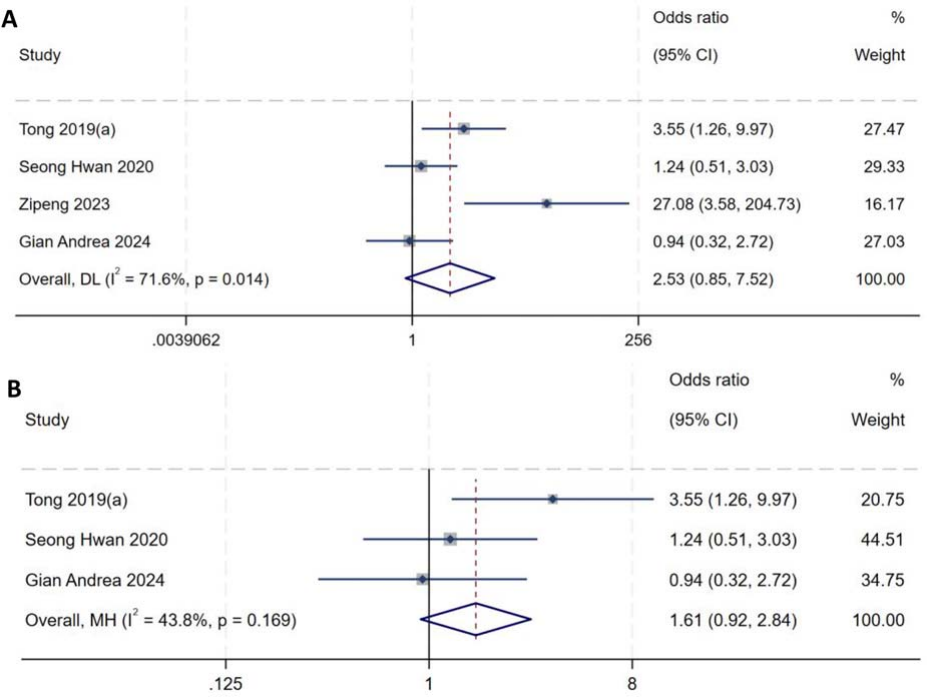
**(A)** Initial analysis with substantial heterogeneity. **(B)** Sensitivity analysis after excluding the study by Zipeng et al., showing the pooled effect size.

A total of four studies (9, 15, 16, 22) were included in the analysis of Lachman test (pivot height). Heterogeneity testing showed an *I*^2^ of 71.6% (*P* = 0.014), indicating substantial between-study heterogeneity, as shown in Figure 8A. A sensitivity analysis with stepwise omission was performed to identify the source of heterogeneity. Exclusion of the study by Zipeng et al. (16) markedly reduced heterogeneity (*I*^2^ = 43.8%, *P* = 0.169). A fixed-effect model was therefore used to pool effect sizes. Meta-analysis demonstrated no significant association between Lachman test (pivot height) and the occurrence of high-grade pivot shift (*OR* = 1.61, 95% *CI*: 0.92–2.84, *P* = 0.10), as shown in Figure 8B.

##### KT-SSD

**FIGURE 9:**
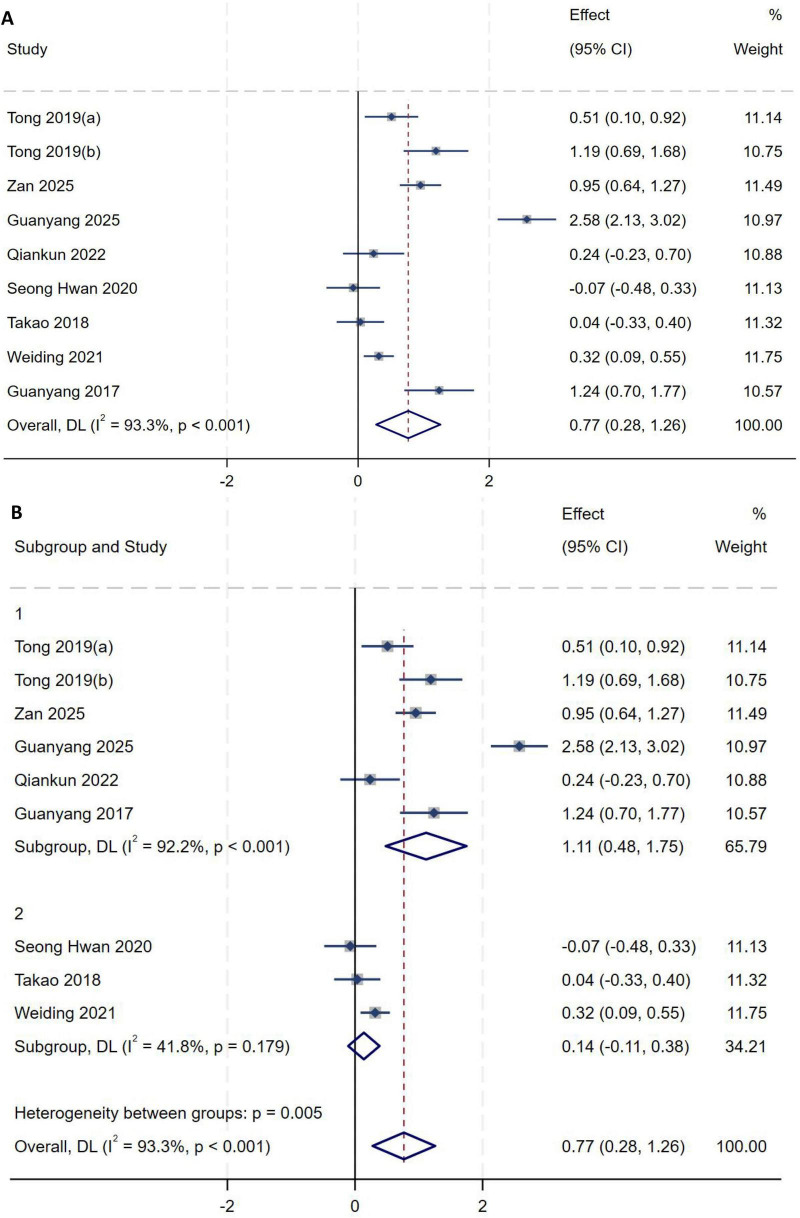
**(A)** Initial analysis with substantial heterogeneity. **(B)** Subgroup analysis by population origin, showing the pooled effect size and reduced heterogeneity.

A total of nine studies (2, 8–10, 14, 15, 19, 20, 23) studies were included in the analysis of KT-SSD. Heterogeneity testing showed an *I*^2^ of 93.3% (*P* < 0.001), indicating substantial between-study heterogeneity. Sensitivity analysis did not significantly alter the overall results. To further explore sources of heterogeneity, subgroup analysis was performed based on study population origin. Six studies (8–10, 14, 20, 23) enrolled participants from China, and the remaining three (2, 15, 19) recruited patients from Korea or Japan. Subgroup analysis was therefore conducted according to whether the study population was from China, with a random-effects model used to pool effect sizes. Meta-analysis demonstrated that increased KT-SSD was an independent risk factor for high-grade pivot shift (*MD* = 0.77, 95% *CI*: 0.28–1.26, *P* = 0.002), as shown in Figure 9A. Studies including non-Chinese populations showed lower heterogeneity (*I*^2^ = 41.8%, *P* = 0.179), indicating that study population origin may represent an important source of heterogeneity, as shown in Figure 9B.

##### KT-SSD (pivot height)

**FIGURE 10:**
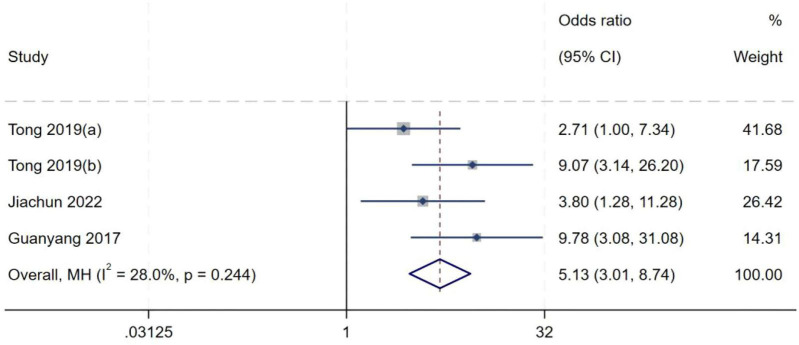
Forest plot of KT-SSD (height) in LMPR+ group versus LMPR− group.

A total of four studies (8, 9, 11, 20) included in the analysis of KT-SSD (pivot height). Heterogeneity testing showed an *I*^2^ of 28.0% (*P* = 0.244), indicating moderate between-study heterogeneity. Sensitivity analysis did not significantly alter the overall results. A fixed-effect model was therefore used to pool effect sizes. Meta-analysis demonstrated that KT-SSD (pivot height) is an independent risk factor for high-grade pivot shift (*OR* = 5.13, 95% *CI*: 3.01–8.74, *P* < 0.01), as shown in Figure 10.

##### Anterior tibial translation (ATT)

**FIGURE 11:**
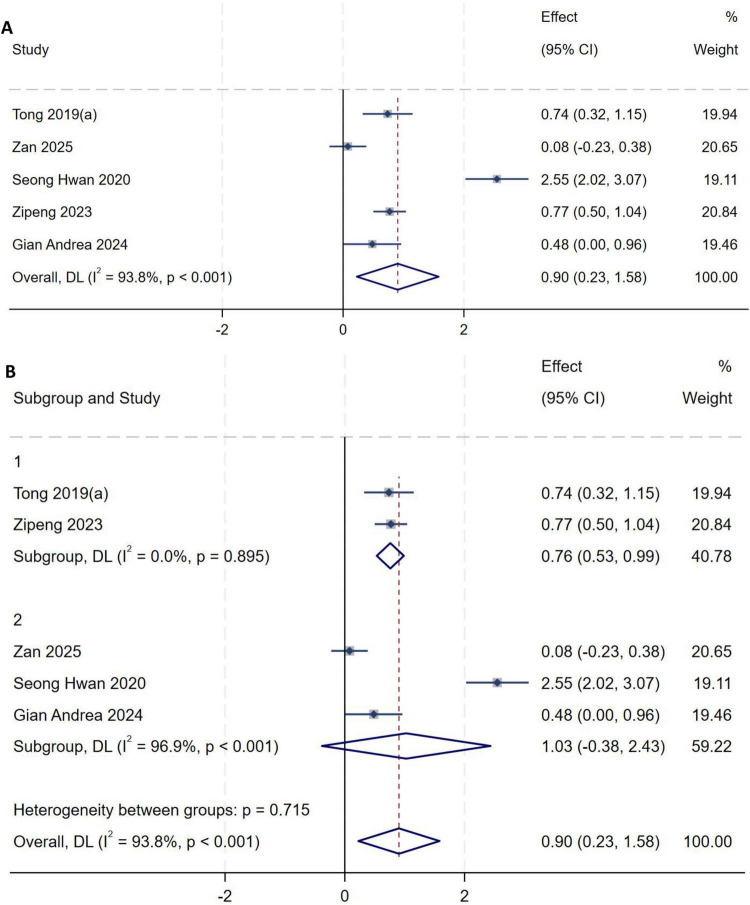
**(A)** Initial analysis with substantial heterogeneity. **(B)** Subgroup analysis by study design, showing the pooled effect size and reduced heterogeneity.

A total of five studies (9, 10, 15, 16, 22) were included in the analysis of ATT. Heterogeneity testing showed an *I*^2^ of 93.8% (*P* < 0.001), indicating substantial between-study heterogeneity. Sensitivity analysis did not significantly alter the overall results. To further explore sources of heterogeneity, subgroup analysis was performed based on study design: among the five included studies, two (9, 16) were case-control studies. Subgroup analysis was therefore conducted using “whether the study design was case-control” as the grouping variable, with a random-effects model used to pool effect sizes. Meta-analysis demonstrated that increased anterior tibial translation (ATT) is an independent risk factor for high-grade pivot shift (*MD* = 0.90, 95% *CI*: 0.23–1.58, *P* = 0.009), as shown in Figure 11A. Studies with a case-control design exhibited significantly lower heterogeneity (*I*^2^ = 0%, *P* = 0.895), indicating that variations in study design may be a key source of the observed high heterogeneity, as shown in Figure 11B.

##### IKDC score

**FIGURE 12:**
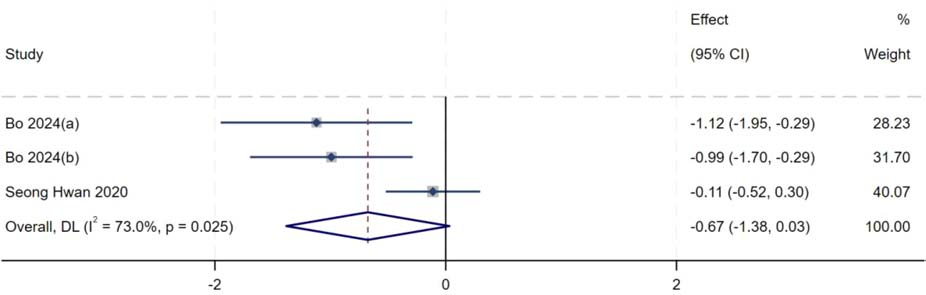
Forest plot of IKDC score in LMPR+ group versus LMPR− group.

A total of three studies (13, 15) were included in the analysis of IKDC score. Heterogeneity testing yielded an *I*^2^ of 73.0% (*P* = 0.025), indicating substantial between-study heterogeneity. Sensitivity analysis did not significantly alter the overall results. A random-effects model was therefore used to pool effect sizes. Meta-analysis demonstrated no statistically significant association between IKDC score and the occurrence of high-grade pivot shift (*MD* = −0.67, 95% *CI*: −1.38–0.03, *P* = 0.061), as shown in Figure 12.

##### Joint laxity

**FIGURE 13:**
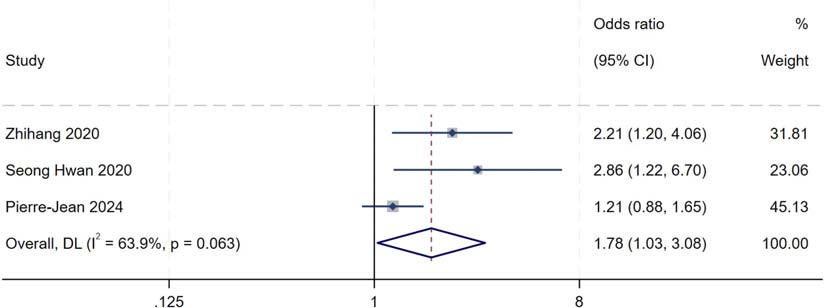
Forest plot of joint laxity in LMPR+ group versus LMPR− group.

A total of three studies (5, 12, 15) were included in the analysis of joint laxity. Heterogeneity testing showed an *I*^2^ of 63.9% (*P* = 0.063), indicating substantial between-study heterogeneity. Sensitivity analysis did not significantly alter the overall results. A random-effects model was therefore used to pool effect sizes. Meta-analysis demonstrated that joint laxity is an independent risk factor for high-grade pivot shift (*OR* = 1.78, 95% *CI*: 1.03–3.08, *P* = 0.04), as shown in Figure 13.

#### Preoperative anatomical risk factors

##### Meniscal extrusion

**FIGURE 14:**
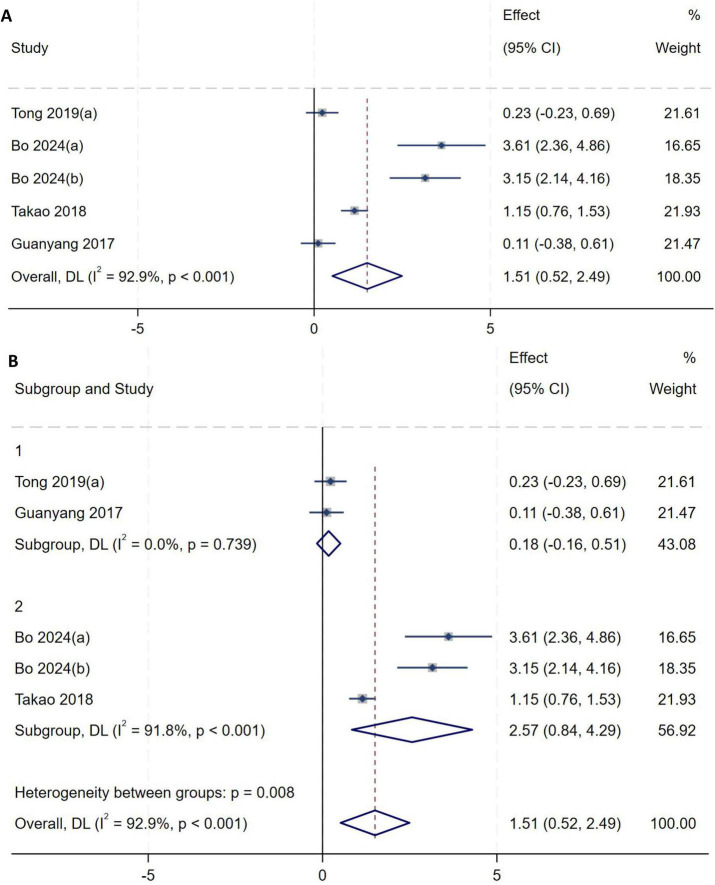
**(A)** Initial analysis with substantial heterogeneity. **(B)** Subgroup analysis by assessment method, showing the pooled effect size and reduced heterogeneity.

A total of five studies (2, 9, 13, 20) were included in the analysis of meniscal extrusion. Heterogeneity testing showed an *I*^2^ of 92.9% (*P* < 0.001), indicating substantial between-study heterogeneity. Sensitivity analysis did not significantly alter the overall results. To further explore sources of heterogeneity, subgroup analysis was performed based on the criteria used to define high-grade pivot shift: two studies (9, 20) utilized imaging-based measurements such as MRI, while the remaining three (2, 13) relied exclusively on physical examination under anesthesia. Subgroup analysis was therefore conducted using the assessment method for high-grade pivot shift as the grouping variable, with a random-effects model used to pool effect sizes. Meta-analysis demonstrated that increased meniscal extrusion is an independent risk factor for high-grade pivot shift (*MD* = 1.51, 95% *CI*: 0.52–2.49, *P* = 0.003), as shown in Figure 14A. Studies using imaging-based methods such as MRI exhibited markedly lower heterogeneity (*I*^2^ = 0%, *P* = 0.739), indicating that the approach used to assess high-grade pivot shift may represent an important source of the observed high heterogeneity, as shown in Figure 14B.

##### Lateral tibial slope of the lateral tibial plateau

**FIGURE 15:**
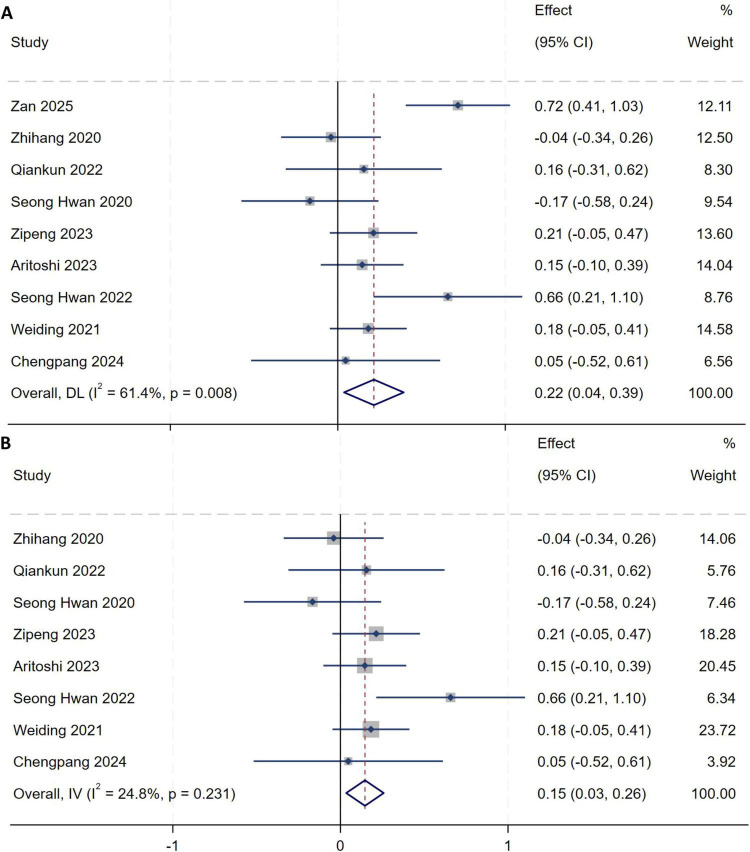
**(A)** Initial analysis with substantial heterogeneity. **(B)** Sensitivity analysis after excluding the study by Zan et al., showing the pooled effect size.

A total of nine studies (10, 12, 14–19, 21) were included in the analysis of lateral tibial slope of the lateral tibial plateau. Heterogeneity testing showed an *I*^2^ of 61.4% (*P* = 0.008), indicating substantial between-study heterogeneity, as shown in Figure 15A. A sensitivity analysis with stepwise omission was performed to identify the source of heterogeneity. Exclusion of the study by Zan et al. (10) markedly reduced heterogeneity (*I*^2^ = 24.8%, *P* = 0.231). A fixed-effect model was therefore used to pool effect sizes. Meta-analysis demonstrated that an increased lateral tibial slope of the lateral tibial plateau is an independent risk factor for high-grade pivot shift (*MD* = 0.15, 95% *CI*: 0.03–0.26, *P* = 0.010), as shown in Figure 15B.

##### Lateral tibial slope of the medial tibial plateau

**FIGURE 16:**
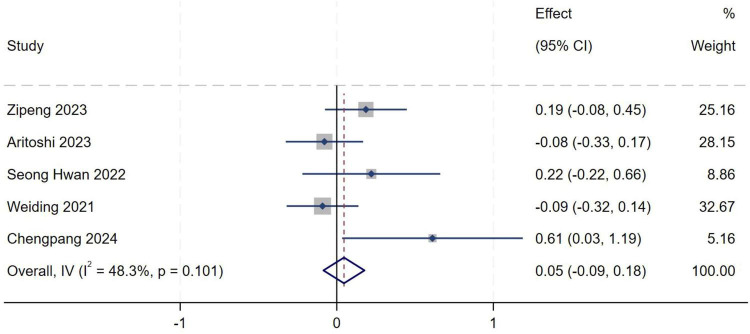
Forest plot of lateral tibial slope of the medial tibial plateau in LMPR+ group versus LMPR− group.

A total of five studies (16–19, 21) were included in the analysis of lateral tibial slope of the medial tibial plateau. Heterogeneity testing showed an *I*^2^ of 48.3% (*P* = 0.101), indicating moderate between-study heterogeneity. A fixed-effect model was therefore used to pool effect sizes. Meta-analysis demonstrated no statistically significant association between lateral tibial slope of the medial tibial plateau and the occurrence of high-grade pivot shift (*MD* = 0.05, 95% *CI*: −0.09–0.18, *P* = 0.492), as shown in Figure 16.

##### Femoral-tibial angle

**FIGURE 17:**
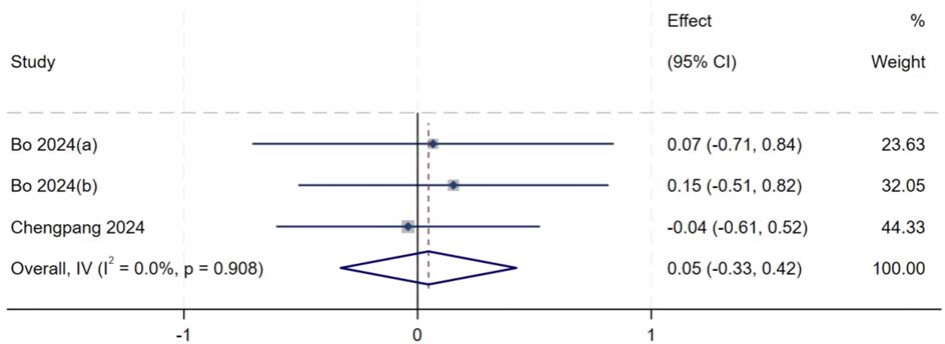
Forest plot of femoral-tibial angle in LMPR+ group versus LMPR− group.

A total of three studies (13, 21) were included in the analysis of femoral-tibial angle. Heterogeneity testing yielded an *I*^2^ of 0% (*P* = 0.908), indicating no significant between-study heterogeneity. A fixed-effect model was therefore used to pool effect sizes. Meta-analysis demonstrated no statistically significant association between femoral-tibial angle and the occurrence of high-grade pivot shift (*MD* = 0.05, 95% *CI*: −0.33– 0.42, *P* = 0.808), as shown in Figure 17.

##### Severe collapse fracture of the posterior margin of the lateral tibial plateau

**FIGURE 18:**
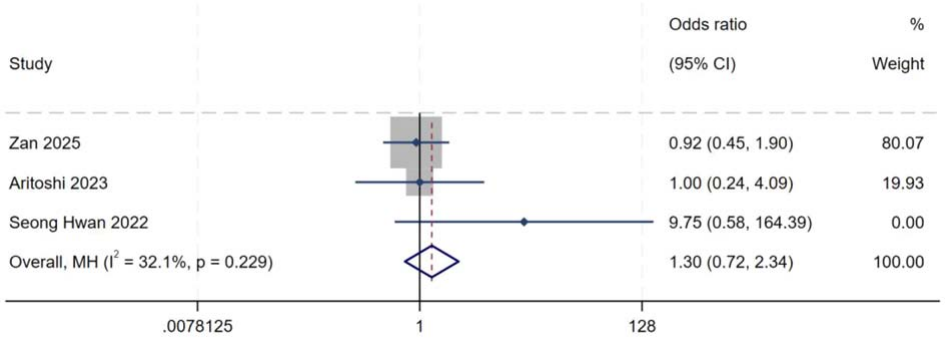
Forest plot of severe collapse fracture of the posterior margin of the lateral tibial plateau in LMPR+ group versus LMPR− group.

A total of three studies (10, 17, 18) were included in the analysis of severe collapse fracture of the posterior margin of the lateral tibial plateau. Heterogeneity testing showed an *I*^2^ of 32.1% (*P* = 0.229), indicating moderate between-study heterogeneity. A fixed-effect model was therefore used to pool effect sizes. Meta-analysis demonstrated no statistically significant association between severe collapse fracture of the posterior margin of the lateral tibial plateau and the occurrence of high-grade pivot shift (*OR* = 1.30, 95% *CI*: 0.72–2.34, *P* = 0.38), as shown in Figure 18.

##### Meniscus-femoral ligament tear

**FIGURE 19:**
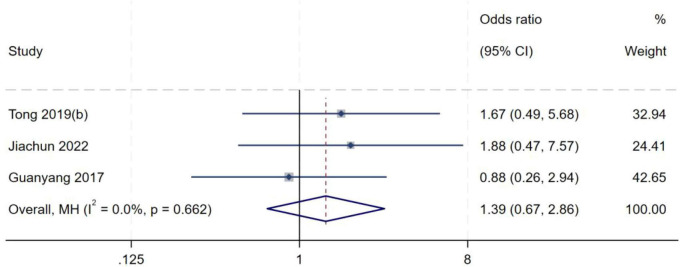
Forest plot of meniscus-femoral ligament tear in LMPR+ group versus LMPR− group.

A total of three studies (8, 11, 20) were included in the analysis of meniscus-femoral ligament tear. Heterogeneity testing yielded an *I*^2^ of 0% (*P* = 0.662), indicating no significant between-study heterogeneity. A fixed-effect model was therefore used to pool effect sizes. Meta-analysis demonstrated no statistically significant association between meniscus-femoral ligament tear and the occurrence of high-grade pivot shift (*OR* = 1.39, 95% *CI*: 0.67–2.86, *P* = 0.377), as shown in Figure 19.

##### Complete meniscus tear

**FIGURE 20:**
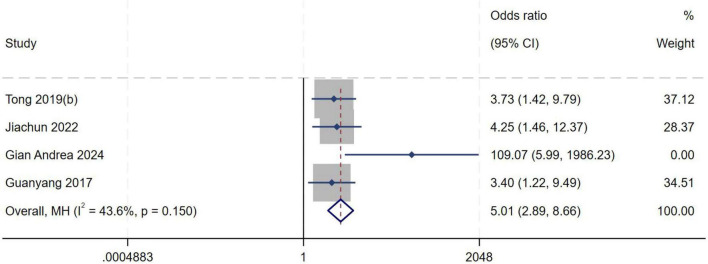
Forest plot of complete meniscus tear in LMPR+ group versus LMPR− group.

A total of four studies (8, 11, 20, 22) were included in the analysis of complete meniscus tear. Heterogeneity testing showed an *I*^2^ of 43.6% (*P* = 0.150), indicating moderate between-study heterogeneity. Sensitivity analysis did not significantly alter the overall results. A fixed-effect model was therefore used to pool effect sizes. Meta-analysis demonstrated that a complete meniscus tear is an independent risk factor for high-grade pivot shift (*OR* = 5.01, 95% *CI*: 2.89–8.66, *P* < 0.001), as shown in Figure 20.

## Discussion

High-grade pivot shift, a key indicator of significant rotational knee instability, strongly influences patients’ preoperative functional status and postoperative rehabilitation outcomes (24). The reported incidence of high-grade pivot shift in patients with ACL tear combined with LMPRT varies widely across relevant studies (5, 9). This meta-analysis found that the incidence of high-grade pivot shift was 72% in patients with ACL tear combined with LMPRT, compared with 46% in those with isolated ACL tear. Sensitivity analysis excluding individual studies did not substantially reduce heterogeneity, suggesting that heterogeneity was not driven by any single study. Further analysis indicated three main sources of heterogeneity. First, regional differences in study populations may represent an important source of heterogeneity. Clinicians from different countries may use different assessment methods and clinical practices, which can affect the evaluation of pivot shift to some degree. Second, variations in study design may also influence the final pooled results, as different designs differ in case selection, bias control, and data collection approaches. Third, studies used different diagnostic criteria for high-grade pivot shift. For example, imaging measurements such as MRI, which have varying sensitivity and specificity across modalities, have been applied in some studies, whereas physical examination under anesthesia has been relied upon solely in others (15, 23). Bias analysis detected no publication bias among the included studies, further supporting the presence of potential multifactorial confounding.

LMPRT leads to a marked increase in the severity of a positive pivot shift, mainly due to its triple detrimental effect on the biomechanical stability of the knee joint (25). The posterior meniscal root serves as a key anchoring structure that maintains meniscal circumferential tension (25). After LMPRT, the meniscus loses its normal load-transmitting function, resulting in complete loss of circumferential tension (25). This causes meniscal extrusion into the joint space, abolishes its constraining effect on the tibial plateau, and impairs its ability to effectively distribute joint loads and restrict abnormal tibial motion (26, 27). Loss of circumferential tension further disrupts the mechanical balance between the medial and lateral knee compartments, leading to a substantial increase in anteroposterior and lateral displacement within the lateral tibial compartment (26, 27). Such abnormal displacement worsens joint malalignment and provides a structural basis for aggravated pivot shift (14). Given that isolated ACL tear already impairs rotational control of the knee, LMPRT further weakens the synergistic function of the knee’s complex stability system, thereby significantly intensifying rotational instability (28, 29). This ultimately manifests as greater tibial displacement and a higher positive grade of pivot shift (28, 29). This biomechanical imbalance forms the core pathophysiological basis for the markedly increased incidence of high-grade pivot shift in patients with ACL tear combined with LMPRT (28, 29).

Furthermore, given the limited number of included studies, this meta-analysis did not conduct a systematic evaluation of several key confounding factors that may substantially influence the severity of knee pivot shift. These factors exhibited substantial variability across included studies and represent a major potential source of heterogeneity in the present findings. First, the time interval from injury to surgery. Patients undergoing delayed surgery are at increased risk of secondary knee injury, with more severe soft tissue scarring and secondary instability that may directly exacerbate pivot shift (30). In contrast, those undergoing acute surgery have relatively uncomplicated soft tissue status, leading to more consistent pivot shift assessments (30). Second, the presence of a Segond fracture. A Segond fracture is a classic radiologic sign of ACL injury with severe rotational instability, indicating concomitant injury to the lateral collateral ligament and posterolateral corner complex (31). Patients with this fracture typically demonstrate more severe pivot shift (31). Third, injury to the anterolateral ligament (ALL). The ALL is a key structure governing knee rotational stability (32). Concomitant ALL injury significantly increases tibial rotational laxity and worsens pivot shift (32). Fourth, generalized ligamentous laxity. Patients with generalized ligamentous laxity have diminished soft tissue tension reserves in the knee (33). Following ACL tear combined with LMPRT, residual knee stability is further compromised, predisposing these patients to high-grade pivot shift (33). Fifth, the surgical management of lateral meniscus root tears (LMPRT). Intraoperative repair of root tears can partially restore meniscal circumferential tension and anchoring function, thereby reducing postoperative pivot shift (34). In contrast, knees without repair (e.g., those treated with debridement or observation) experience more severe loss of meniscal stabilization, resulting in greater pivot shift (34). All these factors can independently alter knee rotational and anteroposterior stability, thereby affecting pivot shift grading. These variables should be recognized as important confounders and study limitations when interpreting the present results.

It is well established that three systems are commonly used to evaluate pivot shift severity: the IKDC pivot shift grading system, the International pivot shift classification, and instrumented pivot shift measurement devices (35). All 20 studies (1, 2, 5, 8–23) included in this meta-analysis used the IKDC pivot shift grading system. Of these, eighteen studies adopted the classic 4-grade scale, defining a grade of ≥ 2 as high-grade pivot shift, whereas two studies (2, 19) used the modified 6-grade classification, with grade ≥ 4 considered high-grade pivot shift.

Currently, there is considerable inconsistency regarding the influence of age on the incidence of high-grade pivot shift in patients with ACL tear combined with LMPRT. No statistically significant association exists between high-grade pivot shift and age (36). In contrast, young age has been reported as an independent risk factor for high-grade pivot shift in this patient population (37). After sequential exclusion of individual studies, sensitivity analysis excluding the study by Zan et al. (10) showed a marked reduction in heterogeneity. Further analysis indicated that the main source of heterogeneity was the substantial differences in surgical interventions between this study and others: while most included studies used standard ACL reconstruction with various graft types and fixation methods, over-the-top ACL reconstruction and extra-articular procedures were employed by Zan et al. (10), which provide more effective rotational stabilization for high-grade pivot shift and may directly influence the incidence of residual postoperative pivot shift. The overall result remained consistent after this source of heterogeneity was addressed via sensitivity analysis, confirming that young age is a robust independent risk factor for high-grade pivot shift in this patient population. This finding suggests that young patients with ACL tear combined with LMPRT warrant greater clinical attention and require comprehensive multidimensional stability evaluation before surgery. However, it is important to clarify that young age may not represent an independent biological risk factor. For younger patients, the observed association with high-grade pivot shift may be partially explained by higher preinjury activity levels, greater participation in high-impact sports, and higher-energy injury mechanisms at the time of ACL tear in this population, which can lead to more severe initial instability and a higher prevalence of concomitant LMPRT (38). Nevertheless, this association is not universal, as it may not apply to sedentary younger patients (38). Therefore, the observed link between young age and high-grade pivot shift is likely mediated by activity level and injury mechanism rather than representing a direct biological effect.

An elevated KT-SSD value is an important risk factor for high-grade pivot shift in patients with ACL tear combined with LMPRT. Rotational laxity under pivot shift loading increases by 23% following LMPRT transection, whereas an intact LMPR can resist 12% of posterior tibial translation (3). The crucial role of the LMPR in posterolateral rotational stability of the knee has also been emphasized (3). LMPRT induces posterolateral tibial rotational instability, which substantially increases anterior laxity and raises KT-SSD by an average of approximately 2.4 mm (39). KT-SSD is a widely used measure for evaluating anterior knee laxity (39). The present study demonstrates that an elevated KT-SSD directly reflects the severity of anterior stability loss and represents an independent risk factor for high-grade pivot shift. Higher KT-SSD values indicate greater anterior tibial translation, and this instability is further amplified by the loss of circumferential tension caused by LMPRT, ultimately manifesting as high-grade pivot shift (39). This finding is consistent with known anatomical mechanisms: the ACL primarily restrains anterior tibial translation, whereas the LMPR, which attaches to the posterior lateral tibial plateau, helps limit tibial rotation and anterior displacement (40). Combined injury results in complex anterior and rotational knee instability (41). This synergistic effect is reflected by increased KT-SSD values, which provide objective preoperative evidence for assessing the risk of high-grade pivot shift (41).

Increased anterior tibial translation is an important risk factor for high-grade pivot shift in patients with ACL tear combined with LMPRT. The findings of this meta-analysis are consistent with several clinical and basic science research results. In an analysis of MRI images from 186 patients with ACL tear, patients with meniscal injuries (including LMPRT) had significantly greater anterior tibial plateau translation (5.7 ± 3.2 mm) than those without meniscal injuries (3.1 ± 2.8 mm) (42). Furthermore, if anterior tibial translation exceeded 6 mm, the incidence of LMPRT increased by 2.7-fold (42). Anterior tibial translation is a key radiologic indicator of anterior knee stability; its elevation essentially reflects the loss of the dual stabilizing functions of the LMPR and ACL (25). Anatomically, the ACL is the primary anterior stabilizing structure of the knee joint, resisting approximately 85% of anterior tibial translation (25). The LMPR, which attaches to the posterior margin of the lateral tibial plateau, assists the meniscus in limiting tibial rotation and anterior translation (25). Particularly in the functional knee flexion range of 30°–90°, the LMPR contributes 12–23% of the anterior stabilizing load (25). After ACL tear, anterior tibial constraint is substantially impaired (43). Concurrent LMPRT eliminates the meniscus’s posterior anchoring effect on the tibia, further increasing anterior translation under pivot shift loading (43). Ultimately, this manifests as a significant elevation in radiologically measured anterior tibial translation values (43).

Current evidence remains inconsistent regarding the influence of joint laxity in patients with ACL tear combined with LMPRT. Joint laxity is an independent risk factor arising from congenital or acquired abnormalities in ligamentous and capsular compliance (44). Patients with joint laxity inherently exhibit reduced tension in the knee’s surrounding supportive structures (44). Following combined ACL and LMPRT injury, their residual stability reserve is completely exhausted, leaving them unable to resist abnormal tibial translation and rotation (44). This significantly increases the likelihood of high-grade pivot shift (44). Clinically, patients with combined injury and joint laxity should receive appropriate preoperative counseling regarding the risk of postoperative instability (45). Postoperative rehabilitation may require a longer duration and more intensive muscle-strengthening training to compensate for deficient structural stability (46).

The present meta-analysis indicates that an increased lateral tibial slope of the lateral tibial plateau represents a significant risk factor for high-grade pivot shift in patients with ACL tear combined with LMPRT. A lateral tibial plateau retroversion angle greater than 12° increases the risk of primary ACL reconstruction failure by 5-fold and revision surgery by 18-fold, representing an independent risk factor for graft failure (47). Patients with a high lateral tibial plateau retroversion angle also have a significantly higher prevalence of meniscal root tears (47). In a 1:1 matched cohort of 103 patients, the lateral tibial plateau retroversion angle was significantly higher in the LMPRT group than in controls (9.1° vs. 7.0°, *P* = 0.001) (48). The risk of LMPRT increased by approximately 1.3-fold for every 1° increase in the lateral tibial plateau retroversion angle (48). The lateral tibial slope is an important anatomical feature that influences knee biomechanics (48). The present meta-analysis demonstrates that increasing lateral tibial slope significantly raises the risk of LMPRT. Biomechanical studies have shown that a steeper lateral tibial slope alters meniscotibial contact mechanics and increases tensile stress within the ACL due to tibial plateau inclination (49). This results in diminished mechanical resistance to anterior tibial translation and rotation (49). In the setting of combined ACL and LMPRT injury, this anatomical abnormality severely compromises joint stability and increases the likelihood of high-grade pivot shift (49). Preoperative imaging measurement of the lateral tibial plateau retroversion angle may guide risk stratification in clinical practice (49). Patients with a markedly increased lateral tibial plateau retroversion angle may require additional stabilizing procedures during ACL surgery, such as meniscal root repair and lateral collateral ligament augmentation (49).

High-grade pivot shift in patients with ACL tear combined with LMPRT is independently associated with both increased meniscal extrusion and complete meniscal tears. In patients with ACL tear, meniscal extrusion of 1.1 mm on MRI predicted posterior root injury with 100% sensitivity and 83% specificity (50). In patients with ACL tear, lateral meniscal extrusion was significantly higher in the complete LMPRT group than in the partial LMPRT and intact groups (*P* < 0.001) (51). Furthermore, the risk of LMPRT increased by 1.7-fold (95% *CI*: 1.2, 2.4) for every 1 mm increase in meniscal extrusion (51). The present meta-analysis shows that increased meniscal extrusion and complete meniscal tears are risk factors for high-grade pivot shift in patients with ACL tear combined with LMPRT. The underlying mechanism is loss of meniscal function (50). Under physiologically healthy conditions, defined by an intact meniscus with preserved circumferential tension, the meniscus resists anterior tibial translation and rotation via its intrinsic load-bearing capacity (50). This function is impaired by LMPRT itself (51). When combined with either meniscal extrusion (which further reduces meniscal stability) or complete meniscal tear (which abolishes its cushioning and stabilizing roles), a triple deficit of intra-articular stabilizers results, severely exacerbating rotational instability (51). Thus, structural abnormalities and loss of core stabilizing function act synergistically to increase the risk of high- meniscal stability grade pivot shift.

This meta-analysis identified several independent risk factors for high-grade pivot shift in patients with ACL tear combined with LMPRT, including younger age, elevated KT-SSD values, increased anterior tibial translation, joint laxity, severe meniscal extrusion, increased lateral tibial plateau retroversion, and complete meniscal tears. Clinically, these factors can serve as preoperative screening indicators to identify patients at high risk of severe rotational instability. Patients with multiple high-risk features require a more comprehensive preoperative knee stability assessment, including detailed evaluation of meniscal status, tibial plateau retroversion, and generalized ligamentous laxity. Regarding surgical decision-making, the findings of this study strongly support concurrent LMPRT repair during ACL reconstruction. LMPRT results in loss of meniscal circumferential tension, increased lateral compartment displacement, and aggravated rotational instability—all of which directly contribute to high-grade pivot shift. Repair of the lateral meniscal posterior root can restore the meniscus’s anchoring function, reduce meniscal extrusion, recover circumferential tension, and simultaneously improve both anterior and rotational knee stability. In patients with marked lateral tibial plateau retroversion, severe meniscal extrusion, or complete root tear, primary root repair is particularly critical, as it minimizes residual pivot shift and reduces the risk of postoperative graft failure and secondary osteoarthritis.

Table 2 summarizes the clinically relevant predictive indicators for high-grade pivot shift. These indicators can be used for risk stratification and intraoperative decision-making. Compared with previous studies, the findings of this meta-analysis are consistent with biomechanical and clinical evidence, namely that lateral tibial plateau retroversion and meniscal extrusion are closely associated with increased rotational instability and more severe pivot shift (29). Consistent with prior biomechanical and clinical studies, the present study confirms that lateral meniscal posterior root tear further compromises meniscal stability, resulting in greater rotational laxity than isolated ACL injury (52). However, this study extends existing knowledge by conducting a quantitative meta-analysis of multiple studies, confirming that the aforementioned anatomical and structural abnormalities act synergistically to ultimately induce high-grade pivot shift.

**TABLE 2:** Clinically relevant predictive indicators for high-grade pivot shift.

Predictor	Type	Clinical significance
Younger age	demographic	Younger patients are more likely to exhibit severe rotational instability.
Increased KT-SSD value	knee anterior laxity	Reflects greater anterior tibial translation and combined instability.
Increased anterior tibial translation	radiological marker	Indicates loss of combined stabilization by ACL and lateral meniscus.
Generalized joint laxity	clinical finding	Reduced soft tissue reserve and worse residual rotational stability.
Increased meniscal extrusion	meniscal status	Loss of meniscal hoop stress and anchoring function.
Increased lateral tibial slope	anatomical factor	Steeper slope increases rotatory laxity and pivot-shift severity.
Complete lateral meniscus root tear	meniscal injury type	Complete loss of posterior root stabilization.

No significant correlation was observed between the incidence of high-grade pivot shift in patients with ACL tear combined with LMPRT and variables such as gender, BMI, Lachman test grade, IKDC score, medial tibial plateau retroversion angle, or femoral-tibial angle. Furthermore, this study has several limitations, which are detailed in Table 3. Future research should adopt well-designed, large-sample, multicenter prospective studies to ensure data representativeness and accuracy. This will help comprehensively explore the mechanisms and associated risk factors for high-grade pivot shift in patients with ACL tear combined with LMPRT while optimizing prevention and intervention strategies.

**TABLE 3:** Limitations of this meta-analysis.

Limitation category	Detailed description
Literature search scope	Only Chinese and English literature were included, with no search of gray literature or relevant databases, which may introduce publication bias.
Geographic generalizability	Only three of the included studies were from Europe and North America, potentially limiting the generalizability of the findings to other regions.
Sample size and result stability	The number of studies investigating specific risk factors was insufficient, which may reduce the stability of the results. Additional high-quality research is needed to validate and refine these findings.
Diagnostic and assessment heterogeneity	There are variations in the diagnostic criteria for LMPRT (MRI vs. intraoperative confirmation) and the assessment methods for high-grade pivot shift (clinical physical examination vs. imaging measurements).
Methodological inconsistency	Measurement methods for several risk factors (e.g., meniscal extrusion values) remain inconsistent, which increases study heterogeneity and may compromise the accuracy of the results.

## Conclusion

Younger age, elevated KT-SSD values, increased anterior tibial translation, joint laxity, severe meniscal extrusion, increased lateral tibial slope, and complete meniscal tear are independent risk factors for high-grade pivot shift in patients with ACL tear combined with LMPRT. Interpretation of joint laxity as an independent risk factor requires caution, given the limited sample size of the included studies, highlighting the need for large-scale, multicenter prospective studies to validate these findings. Nevertheless, this meta-analysis yields valuable clinical implications: enhanced preoperative assessment, optimized surgical strategies, and rigorous postoperative follow-up for patients with ACL tear combined with LMPRT can reduce the incidence of high-grade pivot shift, improve clinical outcomes, and ultimately enhance patients’ quality of life. Patients presenting with these high-risk factors warrant close, enhanced follow-up and timely intervention to mitigate the risk of high-grade pivot shift and optimize long-term patient outcomes.

## Data Availability

The original contributions presented in the study are included in the article/supplementary material, further inquiries can be directed to the corresponding authors.
